# Research note on gravity artifact reduction in IMU signals using Madgwick orientation estimation

**DOI:** 10.1186/s13104-026-08032-w

**Published:** 2026-10-01

**Authors:** Moritz Toppmöller, Urs-Vito Albrecht

**Affiliations:** Department of Digital Medicine, Medical School OWL, Bielefeld University, Universitätsstr. 25, Bielefeld, 33615 NRW Germany

**Keywords:** Orientation, Gravity, Madgwick, IMU

## Abstract

**Objective:**

Gravitational artifacts bias Inertial Measurement Unit (IMU) signals, complicating applications such as Seismocardiography (SCG). This study aimed to validate a gravity-artifact reduction algorithm using Madgwick orientation estimation in a controlled, physics-based simulation.

**Results:**

A computational phantom was developed using the PyBullet physics engine to generate paired datasets of gravity-free acceleration and simulated IMU signals (with gravitational artifacts). The Madgwick Attitude and Heading Reference System (AHRS) filter was applied to estimate the sensor orientation, followed by a low-pass-filtered gravity projection to subtract artifacts. The gravity-removed acceleration closely matched the gravity-free reference across all axes. Absolute mean differences were consistently below 0.006 m/s^2^, and Pearson correlation coefficients exceeded 0.98. Cross-correlation analysis revealed zero temporal lag, while Bland-Altman analysis showed no proportional bias. The highest precision was observed along the gravitational *z*-axis. These simulation-only results represent an upper bound under idealized conditions. Experimental validation remains necessary before practical deployment.

## Introduction

Modern Ballistocardiography (BCG) and Seismocardiography (SCG) rely on Inertial Measurement Units (IMUs) to capture subtle physiological movements. However, accelerometers measure both translational acceleration and a gravitational component. Sensor rotations change their projection onto the individual axes, creating orientation-dependent artifacts that can bias signal interpretation [1].

Accurate orientation estimation helps separate these components. Complementary filters are simple, whereas Extended Kalman Filter (EKF)-based methods require more computational effort and noise-covariance tuning [2, 3]. Direct quaternion integration of gyroscope measurements can suffer from drift without corrective fusion [3, 4]. A prior robotic-arm validation supported the Madgwick filter [5] for its accuracy and computational efficiency, motivating its use for gravity removal [6].

Beyond orientation estimation, accelerometer-only filtering or norm subtraction can separate gravity without gyroscope measurements, but loses accuracy under continuous reorientation [7]. Combined accelerometer–gyroscope approaches estimate orientation and project gravity accordingly, with applications in cardiac motion measurement [8] and smartphone seismocardiography [9]. However, validation through physiological endpoints makes it difficult to establish the accuracy of gravity removal itself.

Obtaining a gravity-free reference from physical IMU measurements remains challenging [4]. To address this, the present study uses a physics-based computational phantom to evaluate the accuracy, temporal alignment, and agreement of the Madgwick-based gravity-removal pipeline [6] against simulated gravity-free acceleration.

## Methods

### Simulation framework

PyBullet [10], a physics engine used in robotics [11], was used to record synchronized angular and linear velocities. The simulation ran in direct mode without Graphical User Interface (GUI) visualization.

### Simulation parameters

**Fig. 1:**
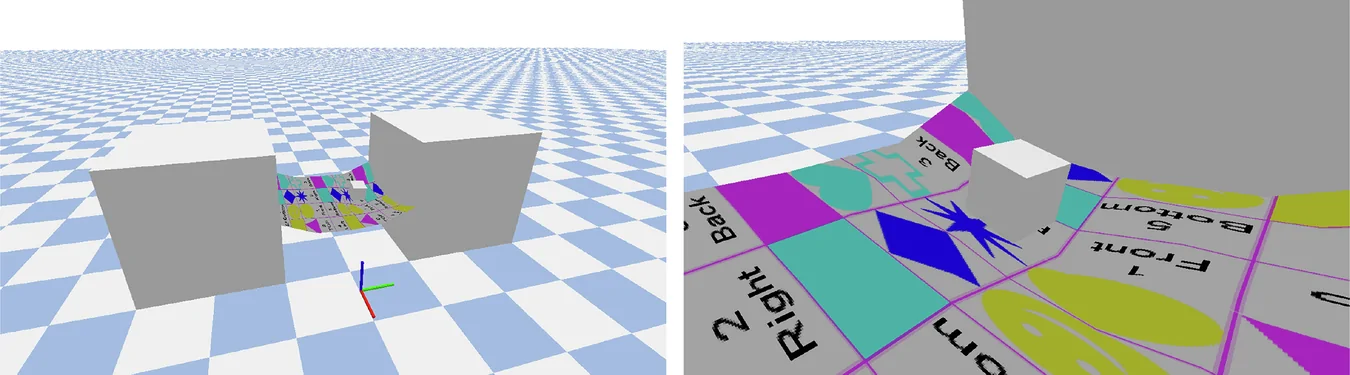
Simulation environment. The left panel shows deformable cloth suspended between two anchor boxes, with the cubic IMU mounting object resting on it and the world coordinate frame shown for reference (x = red, y = green, z = blue). The right panel shows a close-up of the mounting object on the cloth

The simulation ran for 30 s at 1000 Hz, matching sampling rates used in SCG applications [1, 12]. Earth gravity was 9.81 m/s^2^ in the negative world-frame z-direction. A cubic IMU mounting object (0.25 m, 0.25 kg) was dropped onto deformable cloth suspended at its corners between two anchor boxes (Fig. 1). Random external forces were applied to one anchor box during alternating 1 s forcing and resting intervals. This scenario combines non-periodic, multi-axis translation with continuous reorientation, testing gravity removal when both translational acceleration and gravitational projection vary. Scenario selection was constrained by the need to generate physically plausible motion within the physics engine’s numerical limits. The deformable suspension was chosen to provide compliant contact while reducing the risk of numerical artifacts. Peak translational acceleration during analysis was approximately 1.34 m/s^2^. The scenario was not designed to reproduce a specific physiological waveform.

### Sensor modeling

A TDK InvenSense ICM-42688-P [13] IMU was modeled with the following specifications:

- Accelerometer range:  ± 2 g ( ± 19.62 m/s^2^)
- Accelerometer resolution: 16384 LSB/g
- Gyroscope range:  ± 31.25^∘^/s
- Gyroscope resolution: 1048.6 LSB/(^∘^/s)

Raw values were quantized and clipped to the measurable range. No stochastic noise, bias instability, temperature effects, or long-term drift were modeled (see Limitations).

### Signal generation

Linear acceleration was derived from world-frame velocity (*a*_*world*_ = Δ*v*/Δ*t*). The simulated accelerometer signal is obtained by adding gravitational force to the linear acceleration. Since gravity acts in the negative world-frame *z*-direction, this adds a positive force in the *z*-direction. The body-to-world rotation matrix *R* gives

1 $${a}_{truth}={R}^{T}\cdot {a}_{world}$$

2 $${a}_{imu}={R}^{T}\cdot ({a}_{world}+g),\qquad g={[0,0,9.81]}^{T}$$

Additionally, the angular velocity was saved.

### Gravity removal

The Madgwick Attitude and Heading Reference System (AHRS) filter estimates orientation as *q* = [*w*, *x*, *y*, *z*]^*T*^, initialized to *q*_0_ = [1, 0, 0, 0]^*T*^. Orientation is propagated using angular velocity *ω* (rad/s) and corrected using normalized acceleration *a*. The gyroscope-derived quaternion rate is

3 $${\dot{q}}_{\omega }=\dfrac{1}{2}\,q\otimes \left[\begin{array}{c}0\\ \omega \end{array}\right],$$

where  ⊗ denotes quaternion multiplication. Gradient descent aligns predicted and measured gravity directions. The gradient of *f*(*q*, *a*) = q^*^ ⊗ *e*_*z*_ ⊗ *q* − *a* is  ∇ *f* = *J*^*T*^*f*, where *J* is the Jacobian and q^*^ the conjugate quaternion. The combined rate is

4 $$\dot{q}={\dot{q}}_{\omega }-\beta \,\dfrac{\nabla f}{\parallel \nabla f\parallel },$$

where *β* weights the gradient-descent correction relative to gyroscope integration. The filter was used with *β* = 0.05 at 1000 Hz. Orientation is updated by integration and renormalization as

5 $${q}_{t+{{\Delta }}t}={q}_{t}+\dot{q}\,{{\Delta }}t,\qquad {q}_{t+{{\Delta }}t}\leftarrow \dfrac{{q}_{t+{{\Delta }}t}}{\parallel {q}_{t+{{\Delta }}t}\parallel }.$$

The quaternion defines *R*. The gravitational impact factor *f*_*impact*_ is the projection of the positive world-frame *z*-axis unit vector *e*_*z*_ into the body frame,

6 $${f}_{impact}={R}^{T}{e}_{z},\qquad {e}_{z}={[0,0,1]}^{T}$$

The gravity magnitude is estimated from the world-frame vertical acceleration as

7 $${g}_{est}={(R\cdot {a}_{imu})}_{z}$$

A 4th-order Butterworth low-pass filter (cutoff 0.5 Hz, zero-phase forward-backward filtering) isolated the quasi-static gravitational component (*g*_*filtered*_). The gain *β* = 0.05 was adopted from prior validation [6]. Smaller *β* reduces sensitivity to translational acceleration but slows convergence and weakens correction of gyroscope-induced tilt errors. Larger *β* accelerates correction but increases sensitivity to accelerometer disturbances [5]. The 0.5 Hz cutoff suppresses dynamic contamination of the world-frame vertical acceleration. Lower cutoffs smooth this estimate more strongly. Higher cutoffs retain faster variations, allowing more translational acceleration to be mistaken for gravity and subtracted. Cutoff sensitivity is assessed in Sensitivity analysis. Gravity-removed acceleration is given by

8 $${a}_{corrected}={a}_{imu}-{f}_{impact}\cdot {g}_{filtered}$$

### Data processing

Orientation estimation started at 200 ms, after simulation initialization transients. Analysis used simulation times from 3 s onward to exclude filter convergence artifacts [14], leaving 27 s of data.

### Validation

Agreement was assessed per axis using Python 3.14 with NumPy, Pandas, SciPy, and the AHRS library.

#### Error characterization

Mean difference, Standard Deviation (SD), median, and Interquartile Range (IQR) were evaluated. The Signal-to-Noise Ratio (SNR) is

9 $$SNR=10\cdot {\log }_{10}\left(\dfrac{{P}_{{{\rm{signal}}}}}{{P}_{{{\rm{error}}}}}\right),$$

where *P*_*signal*_ is the mean squared gravity-free acceleration and *P*_*error*_ is the mean squared difference between gravity-removed and gravity-free signals.

#### Agreement analysis

Pearson correlation coefficients quantify the linear relationship between gravity-free and gravity-removed signals as

10 $$r=\dfrac{{\sum }_{i = 1}^{n}({x}_{i}-\bar{x})({y}_{i}-\bar{y})}{\sqrt{\mathop{\sum }_{i = 1}^{n}{({x}_{i}-\bar{x})}^{2}}\sqrt{\mathop{\sum }_{i = 1}^{n}{({y}_{i}-\bar{y})}^{2}}},$$

where *x*_*i*_ and *y*_*i*_ are the gravity-free and gravity-removed samples with means $\bar{x}$ and $\bar{y}$. The 95% Confidence Interval (CI) was computed using Fisher’s *z*-transformation as

11 $$z={\mathrm{arctanh}}\,(r),\qquad {{\mbox{SE}}}_{z}=\dfrac{1}{\sqrt{n-3}},\qquad {{\mbox{CI}}}_{95 \% }=\tanh \left(z\pm 1.96\cdot {{\mbox{SE}}}_{z}\right),$$

where *n* is the number of samples. Cross-correlation and the corresponding lag were computed as

12 $${R}_{xy}(\tau )=\mathop{\sum }_{i = 1}^{n}({x}_{i}-\bar{x})({y}_{i+\tau }-\bar{y}),\qquad \hat{\tau }=\arg {\mathop{\max }_{\tau }}{R}_{xy}(\tau ),$$

where $\hat{\tau }$ is the lag at which the maximum cross-correlation occurs.

#### Bland-Altman analysis

The difference *d*_*i*_ = *x*_*i*_ − *y*_*i*_ (gravity-free minus gravity-removed) was computed per sample. Bias and SD of differences were calculated as

13 $$\,{\mbox{bias}}\,=\dfrac{1}{n}\mathop{\sum }_{i = 1}^{n}{d}_{i},\qquad \,{\mbox{SD}}\,=\sqrt{\dfrac{1}{n-1}\mathop{\sum }_{i = 1}^{n}{({d}_{i}-\bar{d})}^{2}},$$

and the Limits of Agreement (LoA) were calculated as bias  ± 1.96 ⋅ SD. The percentage of points outside the LoA and Bland-Altman [15] plots assessed outliers and systematic bias.

#### Distribution analysis

Residual error histograms were used to assess distributional assumptions for the LoA.

## Results

### Signal visualization

**Fig. 2:**
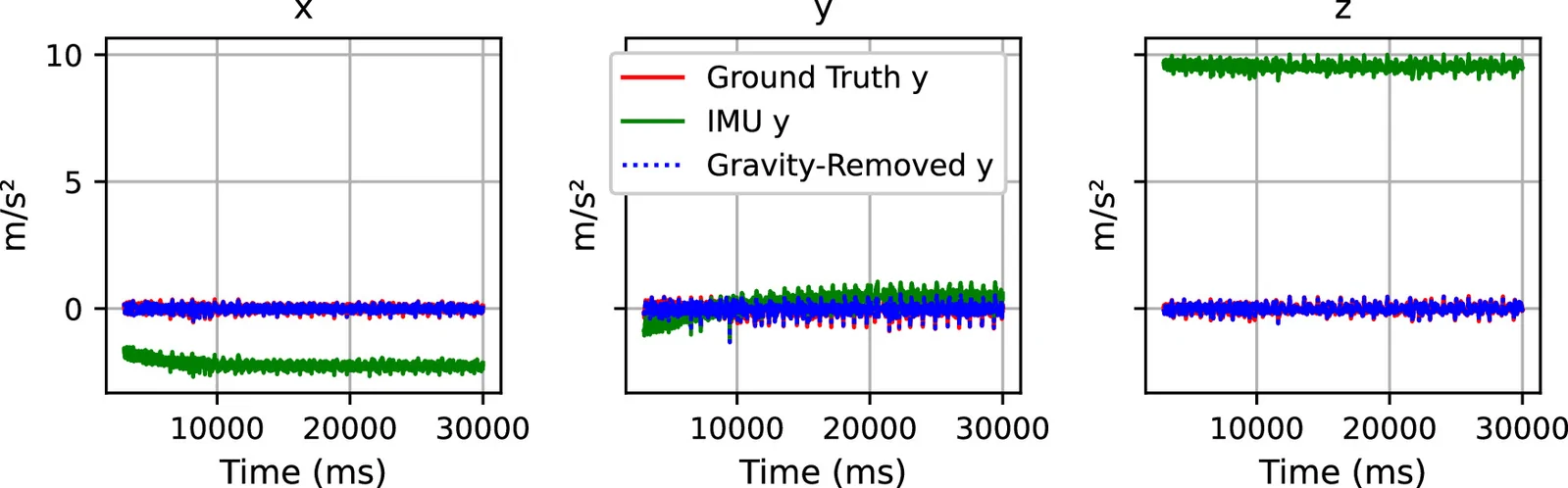
Comparison of acceleration signals along the *x*-, *y*-, and *z*-axes during the analysis window. Each panel shows the gravity-free translational acceleration, the raw IMU signal (with gravitational artifacts), and the gravity-removed estimate

Visual comparison (Fig. 2) showed that gravity removal eliminated the approximately 9.81 m/s^2^z-axis offset, and the gravity-removed signals closely followed the reference. Gyroscope signals were identical between datasets.

**Fig. 3:**
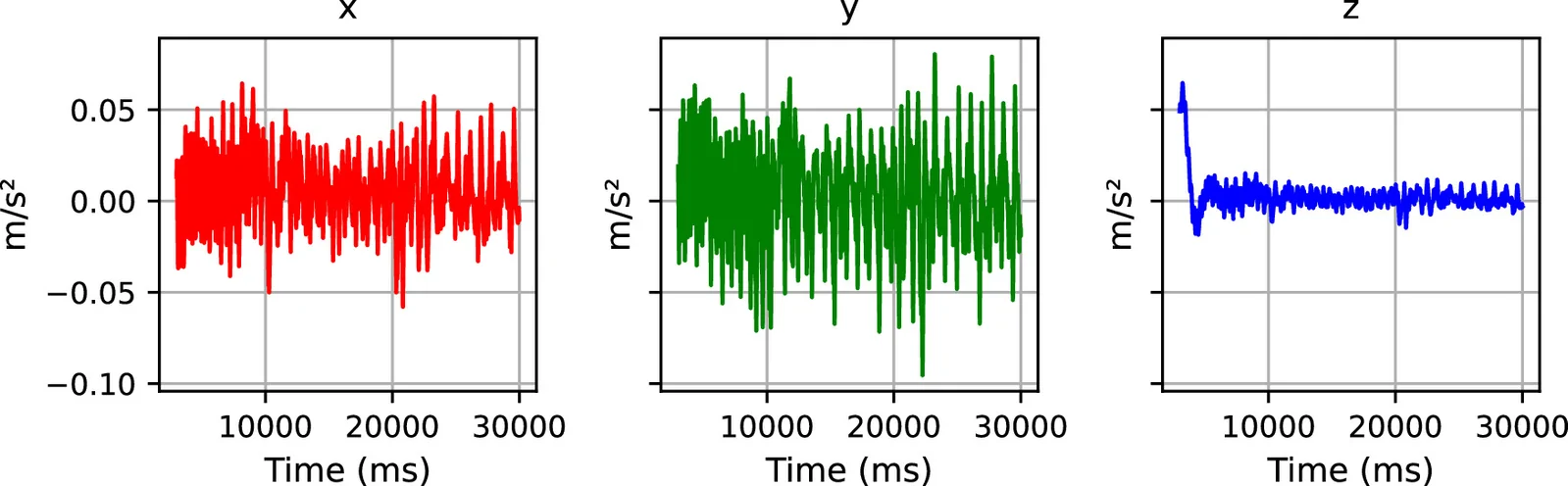
Residual error (gravity-free minus gravity-removed acceleration) for each axis (*x*, *y*, *z*) over the analysis window

Residual errors (Fig. 3) were centered around zero with no systematic drift. Maximum absolute errors were approximately 0.064 m/s^2^ (*x*-axis), 0.095 m/s^2^ (*y*-axis), and 0.065 m/s^2^ (*z*-axis).

### Error characterization

Mean differences ranged from 0.0025 to 0.0055 m/s^2^ (Table 1). The SD and IQR were smallest on the *z*-axis and largest on the *y*-axis. Medians were close to the means.

**Table 1:** Accuracy metrics of the gravity removal algorithm for each body axis, including mean difference, SD, median, IQR, Pearson correlation with 95% CI, SNR, LoA, percentage of points outside the LoA, and cross-correlation lag between gravity-removed and gravity-free acceleration

Axis/Metric	*x*	*y*	*z*
Mean Difference (m/s^2^)	0.0055	0.0030	0.0025
SD (m/s^2^)	0.0194	0.0266	0.0088
Median (m/s^2^)	0.0044	0.0039	0.0012
IQR (m/s^2^)	0.0274	0.0366	0.0060
Pearson Correlation (95% CI)	0.9809 [0.9804, 0.9813]	0.9876 [0.9873, 0.9879]	0.9979 [0.9979, 0.9980]
SNR (dB)	13.8570	16.0367	23.3816
LoA (m/s^2^)	[ − 0.0325, 0.0436]	[ − 0.0492, 0.0552]	[ − 0.0148, 0.0197]
Outside LoA (%)	5.3815	4.9296	3.3037
Cross-Correlation Lag (ms)	0.0000	0.0000	0.0000

### Agreement analysis

Pearson correlation coefficients ranged from 0.9809 (*x*-axis) to 0.9979 (*z*-axis), and SNR ranged from 13.86 to 23.38 dB (Table 1). Cross-correlation revealed zero lag for all axes.

### Bland-Altman analysis

**Fig. 4:**
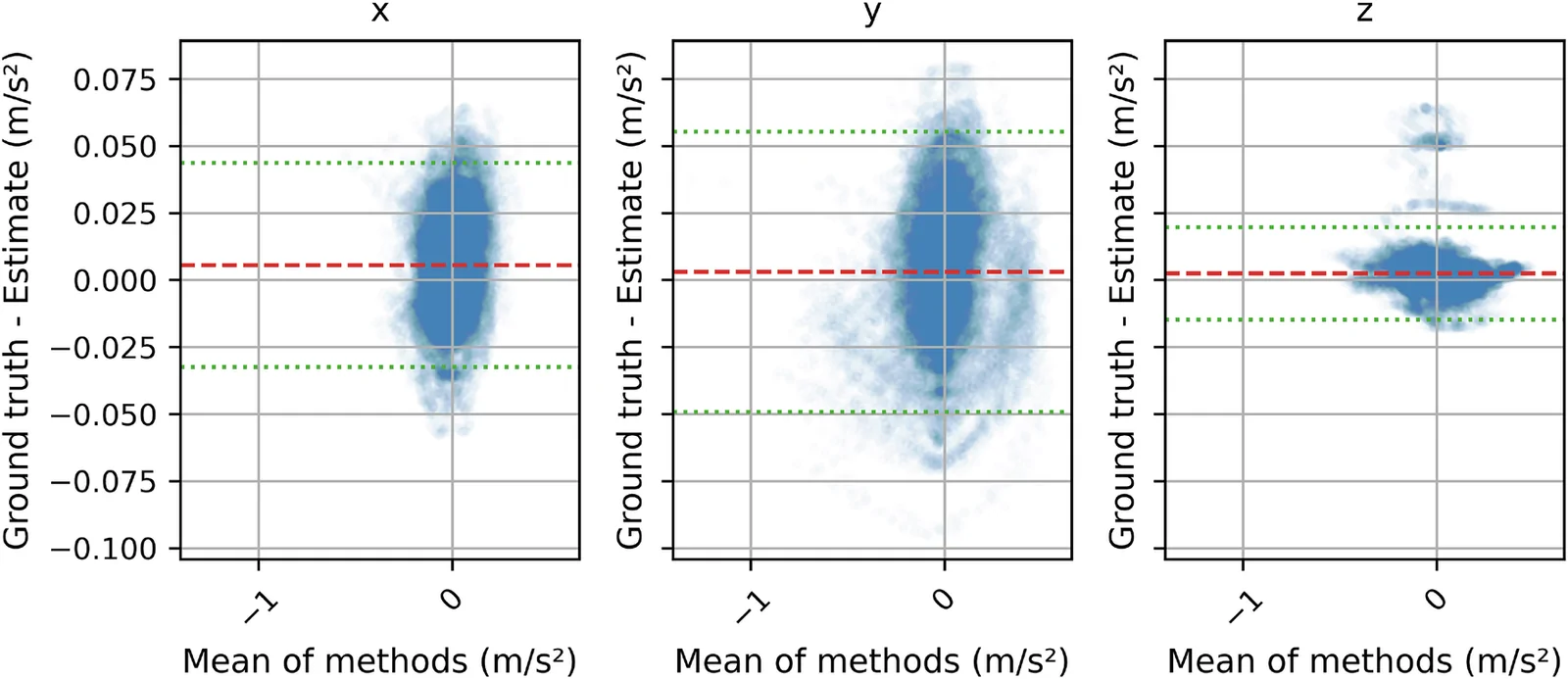
Bland-Altman plots comparing gravity-removed and gravity-free acceleration for each axis (*x*, *y*, *z*). The horizontal axis shows the mean of the two measurements. The vertical axis shows gravity-free minus gravity-removed acceleration. The dashed line indicates the mean difference (bias), and the dotted green lines indicate the upper and lower LoA

Bland-Altman [15] plots (Fig. 4) showed small biases and no evident proportional bias. Points outside the LoA comprised 5.38% (x), 4.93% (y), and 3.30% (z). A cluster of z-axis outliers occurred near zero mean acceleration.

### Distribution analysis

**Fig. 5:**
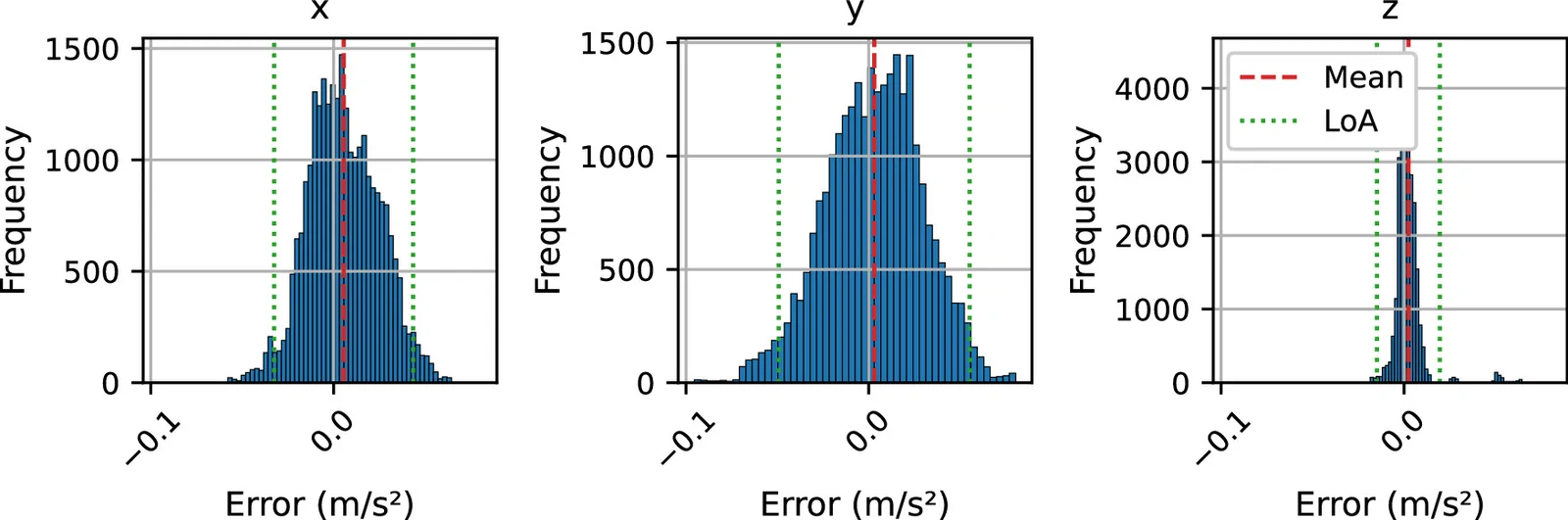
Histogram of the residual errors (gravity-free minus gravity-removed acceleration) for each axis (*x*, *y*, *z*). The red dashed line indicates the mean difference. The green dotted lines indicate the upper and lower LoA

Residual error histograms (Fig. 5) were centered near zero, with wider distributions for the x- and y-axes and a narrower, more peaked distribution for the z-axis.

### Sensitivity analysis

Butterworth cutoff sensitivity was evaluated at 0.1, 0.2, 0.5, 1.0, 2.0, 5.0 Hz with other parameters fixed (Table 2). The 0.5 Hz cutoff yielded a *z*-axis SNR of 23.38 dB, compared with 25.40 dB at 1.0 Hz and 4.35 dB at 0.2 Hz. Thus, 0.5 Hz performed well but was not optimal on every axis. At 2.0 Hz and 5.0 Hz, *z*-axis SNR decreased to 8.49 dB and 2.27 dB, respectively.

**Table 2:** Gravity removal performance for six Butterworth cutoff frequencies, reported per body axis

Cutoff (Hz)	Axis	Mean Difference (m/s^2^)	SD (m/s^2^)	Pearson *r*	SNR (dB)
0.10	*x*	− 0.0050	0.0449	0.9046	6.86
0.10	*y*	0.0002	0.0269	0.9874	16.00
0.10	*z*	0.0549	0.1878	0.5849	− 3.24
0.20	*x*	0.0050	0.0248	0.9685	11.89
0.20	*y*	0.0026	0.0270	0.9873	15.93
0.20	*z*	0.0060	0.0815	0.8564	4.35
0.50	*x*	0.0055	0.0194	0.9809	13.86
0.50	*y*	0.0030	0.0266	0.9876	16.04
0.50	*z*	0.0025	0.0088	0.9979	23.38
1.00	*x*	0.0057	0.0193	0.9812	13.89
1.00	*y*	0.0031	0.0267	0.9876	16.02
1.00	*z*	0.0014	0.0071	0.9986	25.40
2.00	*x*	0.0055	0.0247	0.9688	11.88
2.00	*y*	0.0030	0.0266	0.9876	16.04
2.00	*z*	0.0023	0.0507	0.9289	8.49
5.00	*x*	0.0053	0.0356	0.9350	8.83
5.00	*y*	0.0030	0.0265	0.9878	16.08
5.00	*z*	0.0024	0.1038	0.6489	2.27

## Discussion

The proposed approach, combining Madgwick orientation estimation with low-pass-filtered gravity projection, closely matched the gravity-free reference in this simulation. Residual SD was smallest on the *z*-axis, while cross-correlation showed zero lag across all axes. This temporal alignment is consistent with zero-phase forward-backward filtering (sosfiltfilt) and is relevant to SCG analysis [16].

The low-pass filter complements orientation estimation, as filtering alone cannot distinguish low-frequency translation from changing gravitational projections [7]. The Madgwick filter determines the projection, while the low-pass filter estimates gravity magnitude from the world-frame vertical component before projection into the body frame.

Since validation was performed entirely on simulated data, the reported performance represents an upper bound under idealized conditions. Potential applications in SCG, BCG, wearable monitoring, and robotics require experimental IMU validation before deployment. Complementary work in the ARTIFACTS study compares physical BCG measurements under normal gravity and weightlessness [1], providing a potential route to assess transferability. Future work could use alternative simulation frameworks to explore additional motion scenarios and incorporate realistic sensor error models.

## Limitations

While quantization and clipping were modeled, stochastic noise, bias instability, temperature effects, and long-term drift were omitted. Gyroscope noise and bias would perturb quaternion propagation, whereas accelerometer noise and bias would corrupt the gravity-direction reference for orientation correction. The resulting orientation errors would distort the gravity projection, while vertical accelerometer bias would also shift the filtered gravity estimate, leaving residual artifacts. Temperature effects and long-term drift could introduce time-varying bias, further limiting transferability to physical measurements.

Only one motion scenario and the TDK InvenSense ICM-42688-P sensor model were evaluated, so generalizability to other motions or sensors was not assessed. Neither filter parameter was established as universally optimal: *β* was not varied, and cutoff sensitivity was evaluated only for this scenario. Sustained translation can affect orientation correction, while low-frequency vertical motion can contaminate the gravity estimate. Different motion conditions may therefore require retuning both parameters.

Filter convergence may limit short recordings, while performance during extended recordings remains untested. The non-causal, zero-phase filtering restricts this implementation to offline processing, as real-time use would require causal filtering and separate phase-delay evaluation.

## Data Availability

The datasets generated and analyzed during the current study are available on Zenodounder the following https://doi.org/10.5281/zenodo.21189793 [12]. Additionally, the code is hosted in the following GitLab repository: https://gitlab.ub.unibielefeld.de/ag4-digitale-medizin/papers/2026 bmc-simulation.

## Abbreviations

IMU: Inertial measurement unit
GUI: Graphical user interface
AHRS: Attitude and heading reference system
EKF: Extended kalman filter
SNR: Signal-to-noise ratio
IQR: Interquartile range
CI: Confidence interval
LoA: Limits of agreement
SD: Standard deviation
BCG: Ballistocardiography
SCG: Seismocardiography
